# Global warming is shifting the relationships between fire weather and realized fire-induced CO_2_ emissions in Europe

**DOI:** 10.1038/s41598-022-14480-8

**Published:** 2022-06-20

**Authors:** Jofre Carnicer, Andrés Alegria, Christos Giannakopoulos, Francesca Di Giuseppe, Anna Karali, Nikos Koutsias, Piero Lionello, Mark Parrington, Claudia Vitolo

**Affiliations:** 1 Department of Evolutionary Biology, Ecology and Environmental Sciences, University of Barcelona, Barcelona, Spain; 2 CREAF, 08193 Bellaterra, Catalonia Spain; 3 IRBIO, Biodiversity Research Institute, University of Barcelona, Barcelona, Spain; 4 IPCC Technical Support Unit, Alfred-Wegener-Institute, Bremen, Germany; 5 Institute for Environmental Research and Sustainable Development, National Observatory of Athens, Athens, Greece; 6 European Centre for Medium-Range Weather Forecasts (ECMWF), Reading, UK; 7 Department of Environmental Engineering, University of Patras, Agrinio, Greece; 8 Department of Biological and Environmental Sciences and Technologies, University of Salento, Lecce, Italy; 9 European Space Agency, ESRIN, Frascati, Italy

**Keywords:** Fire ecology, Natural hazards

## Abstract

Fire activity has significantly changed in Europe over the last decades (1980–2020s), with the emergence of summers attaining unprecedented fire prone weather conditions. Here we report a significant shift in the non-stationary relationship linking fire weather conditions and fire intensity measured in terms of CO_2_ emissions released during biomass burning across a latitudinal gradient of European IPCC regions. The reported trends indicate that global warming is possibly inducing an incipient change on regional fire dynamics towards increased fire impacts in Europe, suggesting that emerging risks posed by exceptional fire-weather danger conditions may progressively exceed current wildfire suppression capabilities in the next decades and impact forest carbon sinks.

Climate change will likely produce a widespread change in fire regimes, fire season duration and an increase of the consequent risks across much of the globe over the twenty-first century^1, 2^. A complex picture is already emerging due to the interaction between weather changes and human practices, so while the global trend for burned areas is declining due to socioeconomic factors, some areas of the globe are already experiencing larger and more devastating fires^3^. Particularly, Southern Europe has been long identified as a key hotspot area for risks induced by climate warming, including summer fires, droughts and heat wave events^4–6^. However, increased fire danger projected for this region currently contrasts with realized fire impacts, which have been consistently decreasing over the last five decades mainly due to increasing fire prevention and suppressing capacities, among other complementary factors^6, 7^. Crucially, studies on future fire risk projections indicate that fire impacts in Southern Europe should experience a turning point in the near future, shifting from currently decreasing trends to a high impact trajectory^8, 9^. Overall, due to the large impacts of fire on ecosystem services and multiple social and economic assets, there is an urgent need for more detailed quantitative studies analyzing the changing relationships between fire weather and realized impacts in continental Europe^10, 11^.

Here we address this research goal by analyzing trends of fire danger via utilizing the Fire danger Seasonal Severity Rating index (SSR), a component of the Fire Weather Index system (FWI). The SSR index provides a seasonal average of Daily Severity Rating values (i.e. DSR), which is a nonlinear transformation of the Fire Weather Index (FWI)^12^ (see “Methods” for further details). Fire danger indicators such as the SSR detect dangerous weather conditions conducive to fires rather than modeling the probability of ignition and fire behaviors. In particular the FWI system (developed in Canada) was specifically calibrated to describe the fire intensity in a jack pine stand (*Pinus banksiana*) typical of the Canadian forests^12^. However, its simplicity of implementation has made it a popular choice in many countries, and it has been shown to perform reasonably well in global analyses and in ecosystems very dissimilar to the boreal forest^13–17^. The FWI system is also the rating system for fire monitoring adopted in many European countries and a principal component of the Copernicus Emergency Management Service CEMS^11^. All the components of the FWI system only rely on weather forcings, and neither information on the vegetation status nor on the ignition are taken into account. Thus high fire danger can still be recorded where fires are inhibited due to the scarcity of fuel or as an ignition has not taken place. Despite this limitation, fire danger has been shown to correlate fairly well with fire activity when expressed as burned areas^8, 10, 14, 18, 19^.

In this study historical simulations of SSR for the fire season period are provided by a reanalysis dataset while fire activity is expressed in terms of fire emissions estimated from satellite observations and model simulations^20, 21^. The region of interest is a latitudinal gradient encompassing three IPCC regions (IPCC 2022)^5^ distributed across mainland Europe (Fig. 1a). We start comparing SSR over the last decades (1980–2019) in Southern [SEU], Central [CEU] and Northern [NEU] IPCC regions. As shown in Fig. 1b, SSR has significantly increased in both Southern and Central European IPCC regions (SEU/CEU). As a result, multiple years of high SSR have been recorded over the last decade in summer (Fig. 1b) and spring (Fig. S1). The same qualitative patterns are observed using FWI indices (Fig. S2). As previously mentioned, SSR and FWI weather indices however identify dangerous weather conditions conducive to uncontrollable fires^11^, but do not measure fire activities as they do not consider ignitions, the presence of fuel and fire management practices. It is therefore important to assess whether the elevated SSR levels reached in Southern Europe in the last decade are actually significantly associated with increased impacts in terms of fire emitted CO_2_, shifting in this way the long-term trend of successful suppression of fire in SEU. To detect temporal changes in the relationships between SSR and CO_2_ we applied a moving-window correlation analysis, with a decadal resolution, for the 2000–2020 time period^22^. The main aim of this analysis was to detect non-stationary changes in the changing relationship between fire-prone weather conditions and occurring fires, linked to the recent emergence of years characterized by unprecedented fire events in terms of their impact and number of casualties. The analysis reveals a significant change in the relationship between fire danger and CO_2_ emissions in Southern Europe, progressively shifting from a non-significant relationship to a highly significant linear trend (Fig. 2a,b). In central Europe (CEU), the relationship between fire weather index and biomass burning CO_2_ emissions has remained non-significant (p > 0.05), while in Northern Europe it has significantly changed analogously to Southern Europe (Fig. S3). Fire-induced CO_2_ emissions are significantly lower at higher latitudes (CEU, NEU) over the analyzed period (Fig. S4).

**Figure 1:**
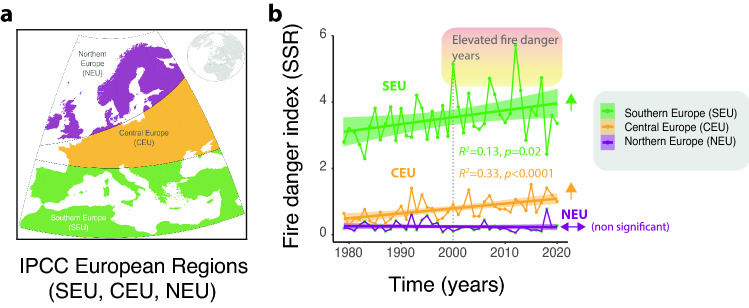
Fire weather dynamics in European IPCC regions. (**a**) A map summarising the distribution of the IPCC regions analysed. The map was produced using QGIS v3.16 (see “Methods”). (**b**) Observed trends in the Seasonal Severity Rating index (SSR) in Southern, Central and Northern Europe over 1980–2019. Colored shaded areas highlight years characterized by higher SSR values over the analyzed period. Ordinary least squares fits are indicated.

**Figure 2:**
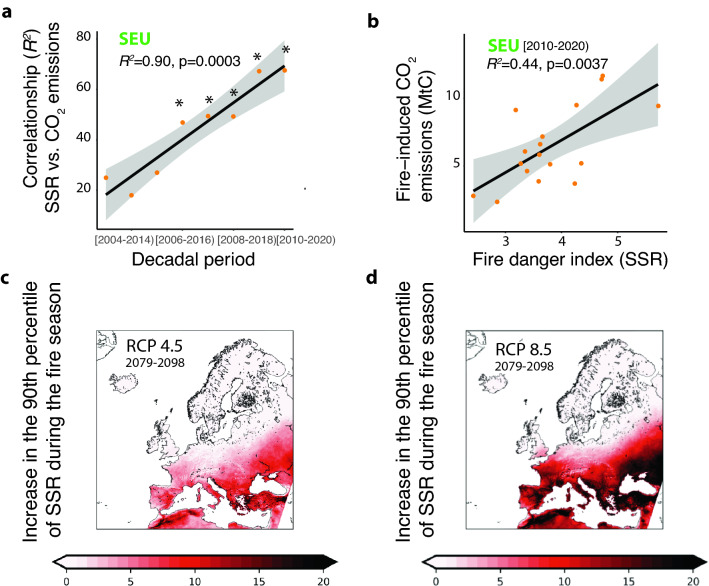
Observed changes in the non-stationary relationships between fire weather danger (SSR) and fire impacts (CO_2_ emissions, MtC) in Southern Europe. (**a**) Changes in the explained variation (*R*^2^) observed in moving-window correlation analyses over the last two decades (2000–2020). Asterisks in panel a (*) indicate significant correlationships, observed only in the last decade. (**b**) Observed relationship between Seasonal Severity Rating index (SSR) and satellite-estimated fire-induced CO_2_ emissions in the last decade. (**c**) Projected increase in the 90th percentile of SSR during the fire season predicted for the 2079–2098 time period under RCP 4.5. SSR increases are represented relative to the values estimated for the reference period (1986–2005, Fig. S4). (**d**) Projected increase in the 90th percentile of SSR under RCP 8.5. Maps were produced using Python Programming Language version 3.8 (see “Methods”).

These findings confirm recent studies indicating that fire regimes can rapidly change in climatic hotspot regions of the globe due to the emergence of non-linear relationships between changed weather conditions and fire frequency and intensity^23–26^. They are also in line with previous results linking SSR, FWI and total burnt area in Europe^14, 18^. Moreover, over the next decades, a further increase in SSR values in this region could be expected. As shown in Fig. 2c,d, under the RCP 4.5 and RCP 8.5 scenarios, extreme fire weather conditions are expected to increase by the end of the century mainly in CEU and SEU. The changes in the 90th percentile of summer SSR show widespread increases of fire danger for both scenarios (Fig. 2). Similar patterns are observed for the spring season, the FWI^90th^ index and the number of days with FWI > 30 (Supplementary Figs. S6–S8). Increases are expected in the frequencies of days with high to extreme conditions (i.e. FWI > 30), up to 20 days per year for SEU and 10 days in CEU under RCP4.5 (Fig. S8B), while the increase will be even higher under RCP8.5, reaching 40 and 30 additional high fire danger days for the same areas (Fig. S8C).

Already burdened areas in SEU, especially for RCP8.5 are projected to face heightened fire danger conditions (Fig. 2d). Crucially, as depicted in Fig. 2c,d, the larger increases expected for SSR will affect geographic areas harboring key carbon sinks of central and southern Europe. For example, larger SSR increases will affect all major mountain range areas of CEU and SEU, including the Pyrenees, Iberian and Cantabrian ranges in Spain, the Alps, Dinaric Alps, the French Central Massif, and the Italian Apennines in central Europe, and the Carpathian, Balkan, Pindus, Caucasus and Pontic Mountains in Southeastern Europe (Fig. 2d, Figs. S5–S7). All these forested areas at increasing fire susceptibility largely contribute to carbon sink ecosystem services in Europe, and are also considered key biodiversity hotspots. The current carbon sink in EU Forests is proximately − 360 MtCO2e/ year, offsetting about 10% of total EU GHG emissions^27, 28^. This carbon sink will likely be progressively reduced by climate change over the XXIth century, due to multiple co-acting processes, including the increased fire risks and associated CO_2_ impacts described (Figs. 1 and 2)^16, 29–31^. Other key co-acting processes include the progressive reduction in CO_2_-induced fertilization effects in forests due to increasing nutrient and water limitations^32, 33^, advancing forest succession and current management practices^28^, increased drought, wind and insect disturbances^22, 34–38^, increased vapour pressure deficit (VPD) and atmospheric evaporative demand effects on forest carbon capture^39–41^, and trade-offs between competing land uses, including food production, forestry and energy uses^42–44^. Despite all these increasing pressures, it has been suggested that counterbalancing fuel management and forestry practices may contribute to reduce the escalating fire risk, and promote carbon capture and resilience in European forest ecosystems^45–48^. Nevertheless, it is widely acknowledged that multiple factors introduce deep uncertainty on the projected trends in fire risks and the fate of forest carbon sinks at risk, including long-term fire-fuel feedbacks and social factors affecting ignition and land uses^6–10, 16, 24, 25^. Beyond fire weather trends, fire regimes are also greatly influenced by human-induced ignition sources, changing fuel management and fire suppression practices, and dynamic changes in land cover and fuels^24, 25, 31^.

Climate change is exerting an increasing control on fire weather, interannual burned area and is progressively changing global fire regimes^24, 49, 50^. The observed changes in non-stationary relationships between fire weather and CO_2_ emissions may be indicative of an incipient change in fire dynamics in Europe (Fig. 2). Previous studies have reported that non-stationary climatic changes significantly affect multidecadal fire dynamics, drought regimes, atmospheric evaporative demand, and largely impact forest carbon sinks in Europe and globally^22, 51, 52^. The reported trends could in turn rapidly imply an increase in fire impacts and emissions over the next decades as has been already reported in other areas of the globe such as the Western United States and Australia^16, 49^. Our results also suggest an incipient change in the current fire regime prevailing in Northern Europe (Fig. S3), consistent with recent assessments pointing to accelerating fire impacts in boreal and arctic regions^53–56^.

Overall, the reported trends suggest an incipient change on fire impacts in Europe. The frequency of heat-induced fire-weather is projected to largely increase in Europe, especially in SEU areas^8, 9^. In line with these findings, in NEU and CEU areas, studies predict an increase in the duration of extreme fire seasons, suggesting that new fire-prone regions in Europe could emerge^57^ (Fig. S3). Despite increasing fire suppression capacities^25, 58^, multiple studies indicate that adaptation limits could be rapidly reached with increasing fire danger levels^6–9, 24, 25, 43^. In line with these reasonings, recent extreme wildfire events in Europe, such as the fires in France (2016), Spain (2017), Portugal (2017), and Greece (2018, 2021), clearly exemplify the limits of wildfire suppression capabilities under exceptional fire-weather danger conditions^9, 25^. Finally, our results also suggest that regional services offering coordinated observations and forecasts of fire weather and fire-induced CO_2_ emissions can facilitate the detection of non-stationary changes in the relationships linking fire weather and impacts, complementing in this way early-warning signal systems of changing fire regimes^50, 56^.

## Methods

Multidecadal trends for the Seasonal Severity Rating (SSR) and Fire Weather Index (FWI) were calculated using the ERA-5 FWI reanalysis dataset “Fire danger indices historical data from the Copernicus Emergency Management Service” available from the Copernicus Data Store (CDS) as gridded data for the period 1980–present^59^. The Seasonal Severity Rating index (SSR) was defined by Harvey et al. (1986) as^60, 61^:

$${\text{SSR}} = \sum {\text{DSR/n}}$$

where *n* is the total number of days considered in the seasonal period, and *DSR* corresponds to the Daily Severity Index^12^, defined as:

$$DSR = 0.0272 \left( {FWI} \right)^{177}$$

Estimates of SSR for Northern Europe (NEU) integrated country-level data for Finland, Norway, Sweden, Denmark, UK and Ireland. The CEU region included France, Belgium, Netherlands, Germany, Switzerland, Austria, Czech Republic, Poland, Slovakia, Hungary, Romania, Ukraine, Moldova, Belarus, Lithuania, Latvia and Estonia. The SEU region included data from Portugal, Spain, Italy, Slovenia, Croatia, Bosnia Herzegovina, Serbia, Montenegro, Kosovo, Albania, Macedonia, Bulgaria, Greece, Turkey and Cyprus. SSR was calculated as the seasonal mean of the monthly spatial averages over each region. The SSR, DSR and FWI equations are generic indices of fire danger^12^. They do not predict where an ignition is likely to occur but rather the meteorological conditions that would cause flames to spread out of control, if an ignition occurred. These conditions, called ‘fire weather’, depend on atmospheric variables such as accumulated precipitation, relative humidity, temperature and wind speed. Since 2007, the FWI has been adopted at the EU level by the European Forest Fire Information System (EFFIS) component of the Copernicus Emergency Management Services, to assess fire danger level in a harmonized way throughout Europe after several tests on its validity and robustness for the European domain^60, 61^. The dataset employed uses weather forcings provided by reanalysis simulations which are created by combining model and quality-controlled observations in a statistically optimal way. A reanalysis provides a dynamically consistent estimate of the climate state at each time step and is to a large extent, be considered as a good proxy for observed meteorological conditions. Projected SSR trends for 2079–2098 period were calculated using C3S Climate Data Store (CDS) data^62^. In the C3S Climate Data Store (CDS) dataset daily FWI values were calculated using the 3-hourly climatic output of a subset of 5 EURO-CORDEX RCM/GCM pairs at a horizontal resolution of 0.11°. To obtain local noon values for index calculations, the 12 UTC climatic output was used as a proxy for the entire European domain^62^. DSR values were calculated from daily FWI values and were afterwards averaged over the period from March to September, the main fire season in Europe according to previous studies^14^.

For projected trends on SSR we assessed the 90th percentile of SSR that corresponds to the upper range of fire danger conditions during the fire season. Long-term changes in the 90th percentile of SSR (2079–2098) were mapped relative to the 1986–2005 reference period under RCP4.5 and RCP8.5. Fire-induced CO_2_ estimates were based on satellite observations of fire radiative power providing a daily global dataset from 2003 to 2019. The estimates of CO_2_ emissions from wildfires were extracted from the Global Fire Assimilation System version 1.2 (GFASv1.2) available from the Copernicus Atmosphere Monitoring Service and European Centre for Medium-Range Weather Forecasts^21^. The GFAS data is based on fire radiative power observations from MODIS instruments on the NASA Terra and Aqua satellites. We applied a moving-window correlation analyses, with a decadal resolution, for the 2000–2020 time period to detect temporal changes in the non-stationary relationships linking fire danger (FWI) and satellite-derived estimates of CO_2_ emissions^22^.

To complement the SSR and FWI analyses, we calculated the trends for Fine Fuel Moisture Code (FFMC) over 1980–2020 in NEU, CEU and SEU regions (Fig. 1). These analyses provided supplementary information on the decadal changes in fine fuel moisture state and the associated ignition risk^12, 26, 63^. For projected risks over the next decades (2079–2098), and to ensure the robustness of the trends reported for the 90th percentile of SSR, we also calculated the annual number of days with FWI > 30 as a complementary approach (supplementary materials) following other relevant climate change impact studies for Europe^64, 65^. FWI > 30 corresponds to high to extreme fire danger based on EFFIS classification for Europe^59, 60, 62^. European IPCC regional maps (Fig. 1a) were produced using QGIS v3.16^66^. FWI/SSR maps were developed using Python Programming Language version 3.8 (Python Software Foundation; http://www.python.org).

## Supplementary Information

Supplementary Information.
