# Supplementary material for: Global warming is shifting the relationships between fire weather and realized fire-induced CO2 emissions in Europe
Source: Sci Rep. 2022 Jun 20;12:10365. doi: 10.1038/s41598-022-14480-8 (PMC9209447; doi:10.1038/s41598-022-14480-8)

Global warming is shifting the relationships between fire weather and realized fire-induced CO_2 emissions in Europe

Supplementary Materials

Supplementary figures

Fig. S1. Observed trends in spring SSR (April-May period) in Southern, central and Northern Europe over 1980-2020. Ordinary least squares fits are indicated. In Southern and Central Europe (SEU, CEU) the variability of SSR values in spring was not significantly correlated with fire-induced CO_2 emissions. For NEU, a significant relationship was observed, which is reported in Figure S3.

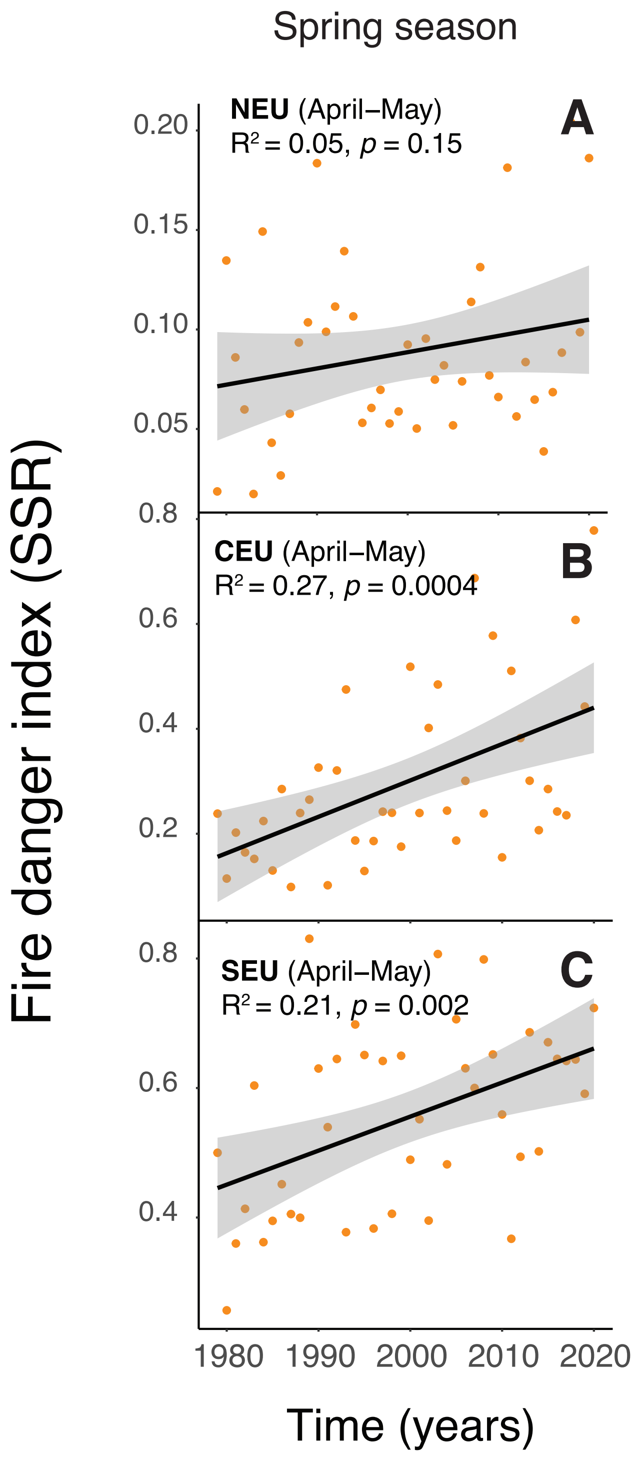

Fig. S2. Observed trends in summer and spring FWI in Southern, central and Northern Europe over 1980-2020. Ordinary least squares fits are indicated.

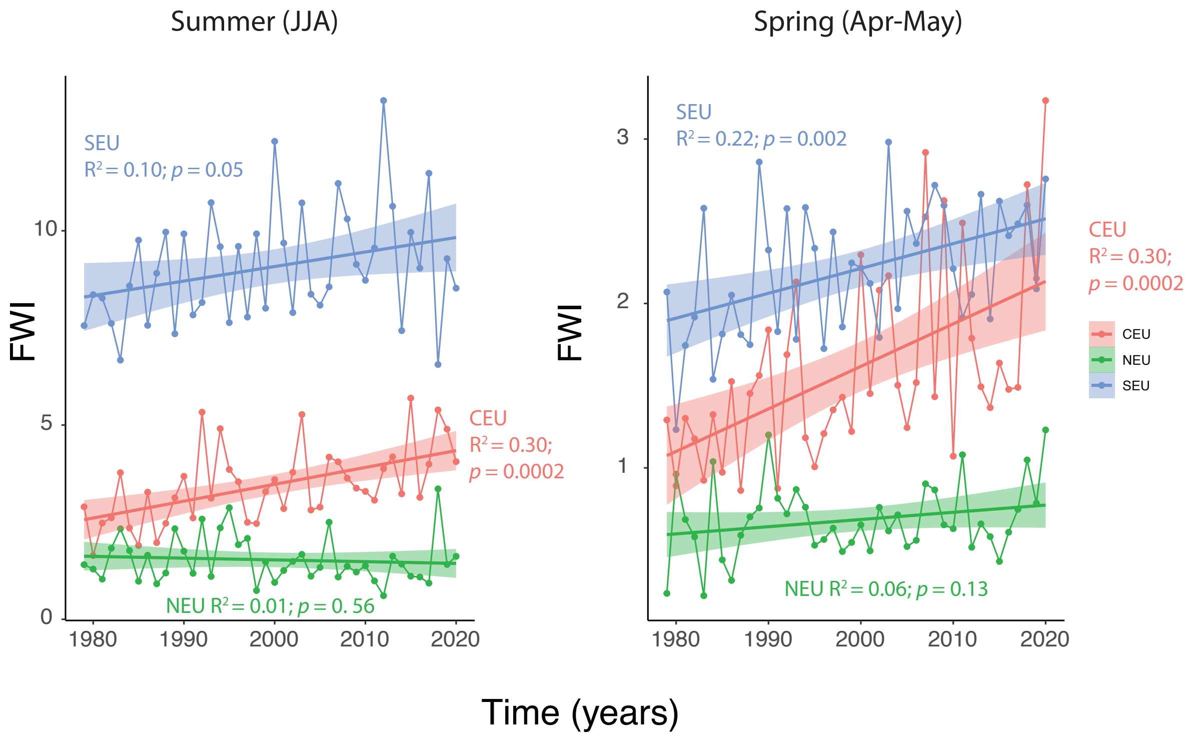

Fig S3. Observed changes in the non-stationary relationships between SSR (April-May) and fire impacts (CO_2 emissions, MtC) in Northern Europe. Changes in the explained variation (R^2) observed in moving-window correlation analyses over the last two decades. Asterisks in panel a (*) indicate significant correlationships, observed only in the last two decades ($p < 0.01$).

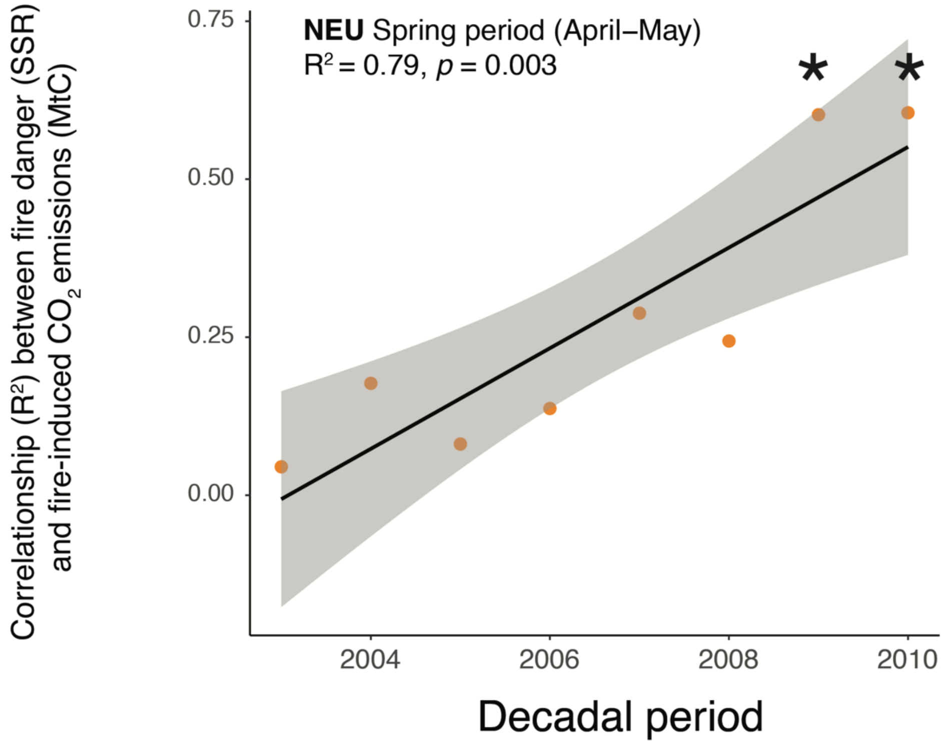

Fig S4. (**a**) Comparison of the observed fire-induced CO_2 emissions for IPCC European regions (NEU, CEU, SEU). Different letters indicate significantly different mean value of weather index and fire emissions, respectively ($p<0.0001$). T-K: a mean comparison Tukey-Kramer test is indicated. **b)** Observed trends on fire-induced CO_2 emissions (MtC) in Southern Europe (SEU), Central Europe (CEU) and Northern Europe (NEU). Dotted lines indicate that all linear trends in the reported temporal series were non-significant in ordinary least squares analyses ($p > 0.05$).

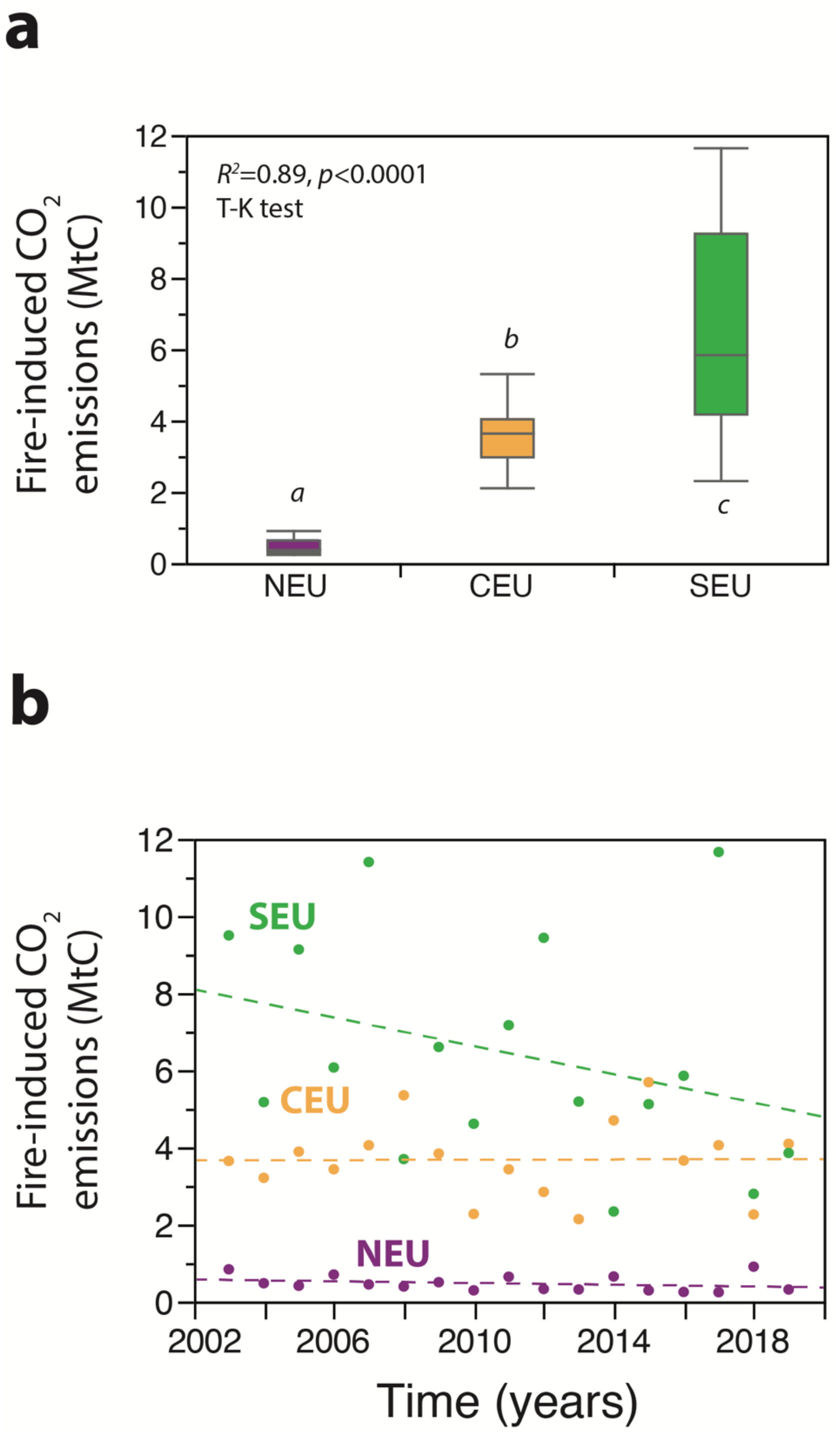

Fig S5. Observed spatial trends in summer 90th percentile of SSR (June-July-August period) over 1986-2005. The figure illustrates the reference period used in Figure 2 to calculate the increase in SSR levels in the 2019-2098 period under RCP 4.5 and 8.5.

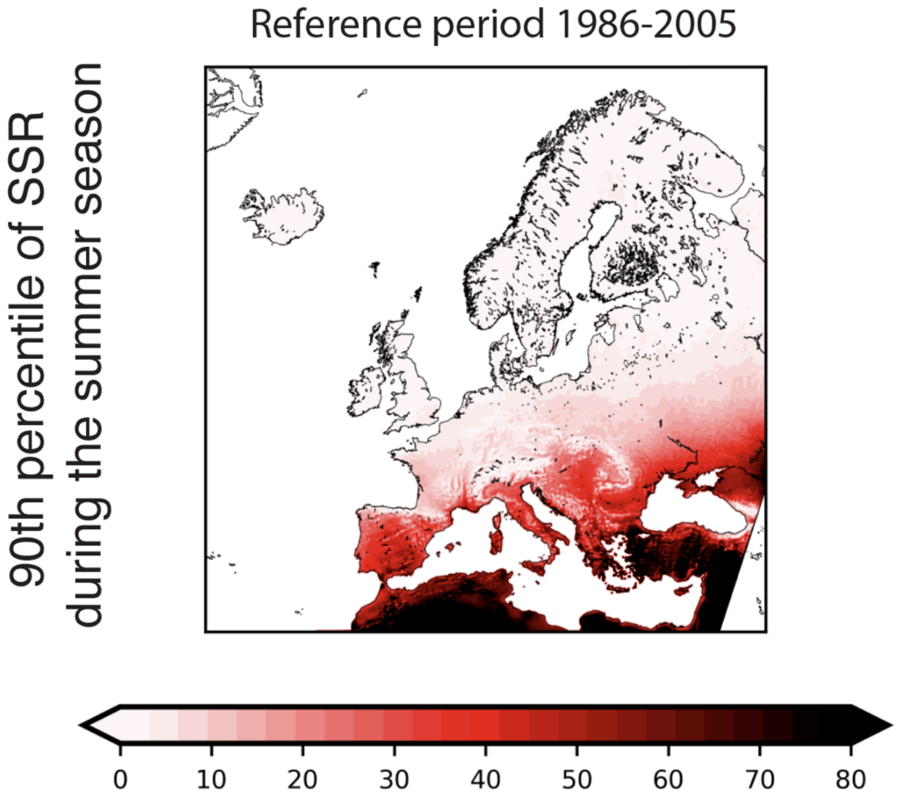

Fig. S6. Projected increase in 90th percentile of spring SSR (April-May period). a) 90th percentile of SSR for the reference period (1986-2005). b) Projected increase in 90th percentile of SSR for the 2079-2098 time period under RCP 4.5. c) Projected increase in SSR under RCP 8.5.

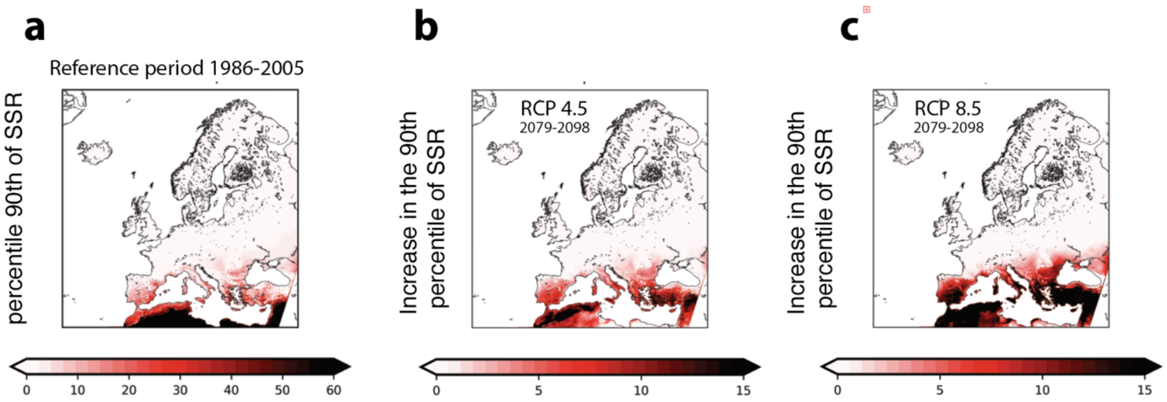

Fig S7. Projected increase in summer 90th percentile of FWI (June-August period). a) 90th percentile of FWI for the reference period (1986-2005). b) Projected increase in 90th percentile of FWI for the 2079-2098 time period under RCP 4.5. c) Projected increase in 90th percentile of FWI under RCP 8.5.

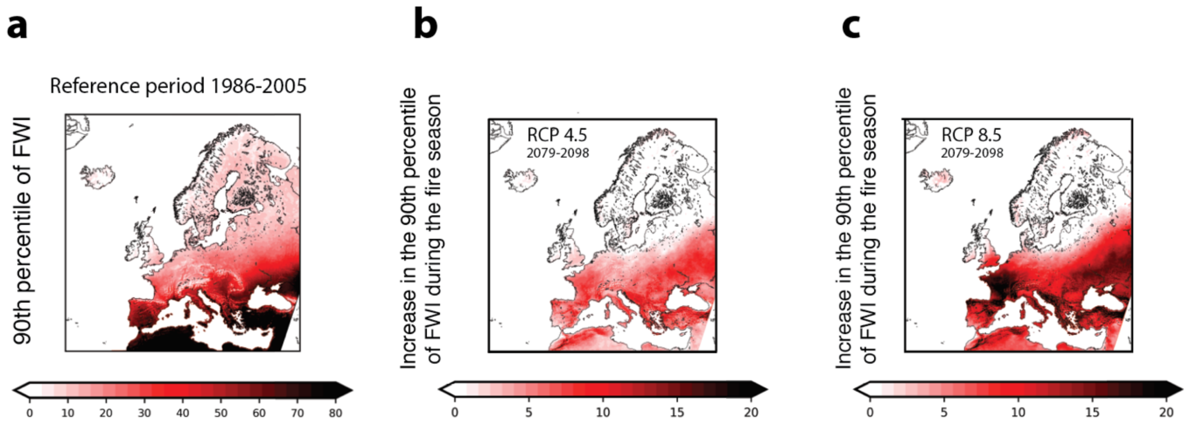

Fig. S8. Projected increase in the annual number of days with FWI>30 (high to extreme fire danger based on EFFIS classification for Europe). a) Number of days with FWI>30 estimated for the reference period (1986-2005). b) Projected increase in the annual number of days with FWI>30 for the 2079-2098 time period under RCP 4.5. c) Projected increase in the annual number of days with FWI>30 under RCP 8.5.

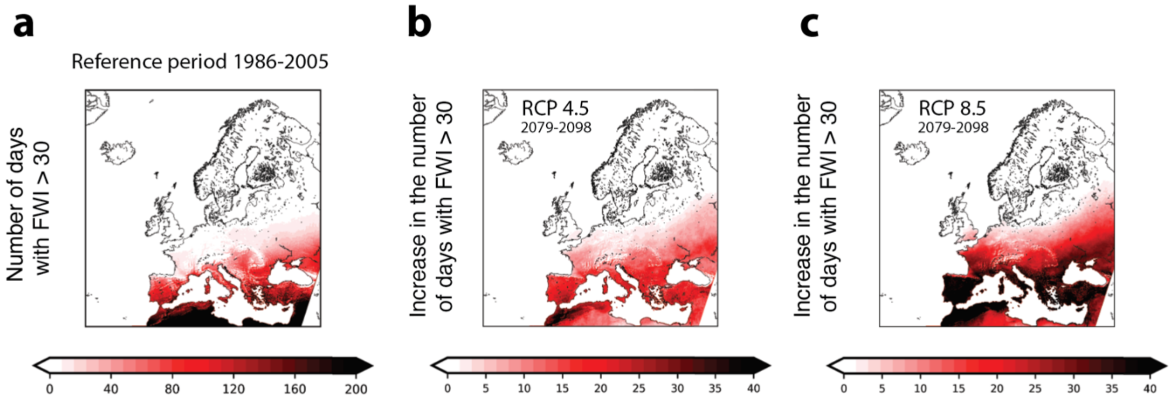

Supplement: Supplementary file 1 — Supplementary Information. [file 41598_2022_14480_MOESM1_ESM.pdf]
